# Supplementary material for: Barriers and perceptions of WHONET/BacLink adoption in Nepal: A qualitative study of clinical microbiology laboratories
Source: PLoS One. 2025 Jul 1;20(7):e0326658. doi: 10.1371/journal.pone.0326658 (PMC12212552; doi:10.1371/journal.pone.0326658)
Supplement: S1 Table — (DOCX) [file pone.0326658.s001.docx]

**S1 Table**. Responses from the participants categorized as per the themes.

| **Themes** | **Sub-themes** | **Responses** |
| --- | --- | --- |
| Support for laboratory staff | Lack of technical support and guidance | - *Technical problem in the system occurred where multiple alerts and warning error signs were displayed on the screen, and we could not use the system then after for about a month-* ***CAPTURA site 7*** - *To move forward, if a technical expert comes and trains the staff properly for 2-3 days regarding the system, then it can be used in the hospital. –* ***CAPTURA site 3*** |
|  | Low literacy and skill in software | - *Information regarding data analysis on WHONET was given. But did not have any idea on how data could be analyzed in WHONET through which methods-* ***CAPTURA site 3*** - *Trainers did not mention their knowledge on the need of uploading the file into the GLASS system and reported that they came to know about it through NPHL training. –* ***CAPTURA site 7*** - *If everyone has the knowledge of data analysis through WHONET, its use will be more consistent in hospitals-* ***CAPTURA site 6*** |
|  | No training or refresher training | - *The two-days training was enough at that time to make us understand but slowly the memory fades away. We forget things and cannot resolve confusion at the time. –* ***CAPTURA site 1*** - *We as trainees could not pass on the knowledge on how to use WHONET to other staffs. We ourselves are unconfident about the system. So, if trainers were to come on site and give training for 2-3 days it would be more fruitful. -* ***CAPTURA site 4*** |
| System within hospital | Less involvement of hospital management | - *Everyone at authoritative positions at hospital are busy with their own task and no efforts has been made to enhance the existing knowledge and use of WHONET. Neither has the management team put effort nor individuals receiving training has taken a lead. -* ***CAPTURA site 7*** - *It would be better if someone from the IT department also participated in the training. In case any problem arises, we can seek help from the IT staff as well. –* ***CAPTURA site 4*** - *Lack of support from respective experts and management. No discussion with the staff has been held regarding the implementation of new software or brining another system. –* ***CAPTURA site 3*** |
|  | Poor system integration and interoperability | - *The data once entered must be entered again in the lab for Midas. Emergency, OPD, ICU these departments have their data linked while on the other and WHONET is not linked throughout the whole hospital. –* ***CAPTURA site 3*** - *WHONET is very useful for data analysis, but it is not effective for the hospital. This is because the hospital software works separately from the WHONET, and it is not linked with other departments within. Use of WHONET system alone would demand double entry of data in the two systems which is time consuming. –* ***CAPTURA site 4*** |
|  | Poor capacity building approach | - *We do not feel confident enough to use the system independently. Extracting data and using it again through backlink is still difficult for us.* ***– CAPTURA site 4*** - *Lack of confidence in using WHONET system is due to the gap that exists between training and practice duration. –* ***CAPTURA site 1*** |
|  | Resources constraints | - *Availability of single staff who knows how to use WHONET so in absence of that staff, no one else can enter data. -* ***CAPTURA site 5*** - *Staff for entering lab data is available but in the microbiology department there are only two staff. I handle most of the data and have taught the other staff as well to enter data in WHONET, but besides that I am responsible for everything. –* ***CAPTURA site 2*** |
| WHONET and BacLink system in hospital | Impact of WHONET training | - *The training was effective content and methods wise. PowerPoint presentation and real time data demonstration was done using the hospital data as dummy data for WHONET in training. However, the duration of training was insufficient. Two days training is not sufficient. We did not have time to discuss with trainers, on the real problem that we faced while using it in hospital setting for real. –* ***CAPTURA site 7*** |
|  | Lack of user's involvement | - *Lack of motivation in hospital workers/lab officials to conduct their own data analysis is seen at hospital. No one takes the initiative. –* ***CAPTURA site 6*** - *We have very little knowledge about backlink so do not use it all. We need codebooks to acquaint new recruits with how to use the software. –* ***CAPTURA site 2*** - *Need more initiation in terms of approaching the management team for implementation of WHONET. –* ***CAPTURA site 1*** |
|  | Problem reaching to patient's end | - *Report printing from WHONET software was not in good format. Thus, do not use it for report printing. –* ***CAPTURA site 2*** - *WHONET does not allow other users except the assigned ones to access the patients' details, or reports and also the printed report from WHONET is not intelligible. This issue was put forward but not addressed in any of the trainings-* ***CAPTURA site 6*** |
|  | System usability and practice | - *During the training it seemed to be useful in terms of microbiology aspects and looking into bacteria. But after the training we have never been able to put the software to use, though we look forward to it. –* ***CAPTURA site 10*** |
| Data use and analysis | Less practice of data analysis and reporting | - *The WHONET training was good, but we were not able to utilize it properly. Right now, we just enter data into our system and print reports but if we had used WHONET we would have been able to analyze data for a year or two years and make reports. -* ***CAPTURA site 1*** - *No antibiotic alerts or breakpoints have been set in the laboratory. AMR pattern identification is not practiced. Only the RIS mapping is done for each antibiotic against the organism on a periodic basis. –* ***CAPTURA site 7*** |
|  | Less knowledge on system usability | - *Since we had started directly uploading data into WHONET, the backlink was never put into use. So, we did not deal with the system. The confusion lies in using backlink. –* ***CAPTURA site 4*** - *Information regarding data analysis on WHONET was given. But did not have any idea on how data could be analyzed in WHONET through which methods-* ***CAPTURA site 3*** |
