# Supplementary material for: Barriers and perceptions of WHONET/BacLink adoption in Nepal: A qualitative study of clinical microbiology laboratories
Source: PLoS One. 2025 Jul 1;20(7):e0326658. doi: 10.1371/journal.pone.0326658 (PMC12212552; doi:10.1371/journal.pone.0326658)
Supplement: S2 Table — (DOCX) [file pone.0326658.s002.docx]

**S2 Table.** Post-training assessment interview guideline

| 1. **About WHONET/BACLINK training (content; appropriateness; effectiveness)**  - How do you describe the WHONET/BacLink training? (Probe: How was the content? How was the trainer? How many participants were there?) - How appropriate did you find the content of the training? (Probe: For hospital setting, laboratory setting, for microbiological and clinical data management) - How effective was the overall training? (Probe: In terms of applicability, duration and methods use, participants selected. How has the training been helpful in bringing change in data recording and management system at hospital?) - What system was in place for AMR related data collection and storage before training? Now? (Probe: What impacts did you observe/feel following the training? In terms of data; changes in system of data collection, advantages, and disadvantages of existing system) |
| --- |
| 1. **Implementation experiences (duration of implementation; perceived benefits**)  - Are you implementing the WHONET/BacLink software in your hospital?   *If yes,*   - How long have you been implementing the practice of WHONET/BacLink software? - For what purpose are you using the software? (Probe: data entry; Data analysis; Report Preparation and dissemination) - What benefits have you felt after the implementation of this software? (Probe: data entry and analysis; reporting; AMR pattern identification, alerts)   If no,   - Since when did you stop the implementation? (Probe: did not start at all; stopped in the middle) and why? - Why have hospitals not implemented the software? (Probe: lack of trained human resources; difficulty in implementation; felt convenient to use previously used LIS or traditional system; insufficient training; lack of supervision or follow-up, resource drain, budget, reporting and referral problem) |
| 1. **Barriers to implementation (problems and challenges; mitigation measures**)  - What problems/barriers have you faced while implementing the software? (Probe: Technical problems; lack of guidance/go-to person during problems; lack of trained human resources, managerial problem, financial problem) - How have you been mitigating these challenges? (Probe: following the WHONET/BACLINK guide; getting support from trainers; seeking solutions online) |
| 1. **Suggestions: willingness to continue; way forward**  - To sum up, how do you describe the usefulness of training and WHONET/BACLINK? - What suggestion would you like to give to the training team? - How do you explain your willingness to continue the use of software? (Probe: if not using the software, are there ways that can motivate you to use the software?) - How can the software be improved and customized as per the need of the hospital? |
