# Supplementary material for: Barriers and perceptions of WHONET/BacLink adoption in Nepal: A qualitative study of clinical microbiology laboratories
Source: PLoS One. 2025 Jul 1;20(7):e0326658. doi: 10.1371/journal.pone.0326658 (PMC12212552; doi:10.1371/journal.pone.0326658)
Supplement: S3 Table — (DOCX) [file pone.0326658.s003.docx]

**S3 Table**. Summary table on implementation barriers and possible solutions

| Implementation barriers | Possible solution |
| --- | --- |
| - Unavailability and insufficient staffs to provide technical support in promoting and strengthening the usability of system | - Recruit additional technical personnel dedicated to providing ongoing support and troubleshooting for system-related issues. - Collaborate with the system provider to establish regular workshops or webinars specifically tailored to the hospital's needs for continuous education. - Implement comprehensive training programs for existing staff to increase their technical proficiency in utilizing the system effectively. - Establish a helpdesk or support hotline within the hospital premises for immediate assistance with system-related queries or problems. |
| - Insufficient training and refresher training on WHONET/BacLink | - Organizing and arranging the training and refresher training to the staffs recruited for technical support. - Encourage a culture of continuous learning and adaptation by organizing periodic refresher courses or knowledge-sharing sessions. - Develop detailed documentation or manual for the system's usage and troubleshooting, easily accessible to all staff members. - Develop easily accessible online training modules or resources for continuous self-learning. - Organize periodic workshops or seminars focused on advanced features and best practices of the WHONET/BacLink system. - Establish a mentorship program where experienced users can guide and support new or less proficient staff members in navigating the system effectively |
| - Reduced interest among admin and management personnel on setting up and introducing WHONET/BacLink system | - Engage in one-on-one discussions or meetings with administrative and management personnel to address their concerns and emphasize the system's potential to streamline operations and enhance data management. - Develop a clear and compelling business case outlining the return on investment, efficiency gains, and improved data-driven decision-making facilitated by the system. - Seek endorsements or testimonials from influential stakeholders or key opinion leaders within the healthcare industry advocating for the adoption of WHONET/BacLink. - Establish pilot programs or trials within specific departments or units to demonstrate the system's efficacy in a controlled environment before full-scale implementation. |
| - Lesser use of data stored in WHONET system for case analysis | - Provide additional training specifically focused on data analysis tools and techniques within the WHONET system to improve staff proficiency. - Create user-friendly dashboards or interfaces that facilitate easy access and interpretation of data stored in the WHONET system. - Encourage interdisciplinary collaboration among healthcare professionals to promote the utilization of WHONET data for case analysis and decision-making. - Develop standardized protocols or guidelines for utilizing WHONET data in case analysis to ensure consistency and encourage its regular use. - Offer incentives or recognition programs for departments or individuals who demonstrate effective use of WHONET data in conducting case analyses leading to improved patient outcomes |
