# Supplementary material for: Barriers and perceptions of WHONET/BacLink adoption in Nepal: A qualitative study of clinical microbiology laboratories
Source: PLoS One. 2025 Jul 1;20(7):e0326658. doi: 10.1371/journal.pone.0326658 (PMC12212552; doi:10.1371/journal.pone.0326658)
Supplement: S4 Table — (DOCX) [file pone.0326658.s004.docx]

**S4 Table**. De-identified data supporting the findings of this study

| **About WHONET/BacLink training** |  | |
| --- | --- | --- |
| - Has been using WHONET at Site 1 since 2014 and had prior experience with it even before the training. However, following the training, significant improvements were made in its application within the hospital setting. - Before the training, WHONET was used solely for data entry and recording process at the hospital, as they were unaware of how to extract and upload available data in different formats into the system. Instead, WHONET was limited to recording, while Excel was used for analysis and other applications. - After the training, the introduction of BacLink enabled system users to extract and upload files in various formats, streamlining the process. Additionally, the training improved their ability to analyze data and generate reports for submission to clinicians. | 25^th^ July 2022 Interviewee 1 Site 1 | |
| - A total of five participants, purposively selected by the hospital management team, attended the training. However, due to a busy schedule in the hospital laboratory, only three participants could take part in the training on analysis. The training was conducted exclusively for the microbiology team. - No in-house training was conducted. - Currently, only one person is using the system for data entry and analysis. One staff member has left, while another has been assigned to a different department. - Overall, the training experience was beneficial and valuable, even though they had been using WHONET from the beginning. BacLink was new to the team and proved to be particularly useful, as it allowed them to clean the data once a month and upload it to WHONET efficiently. Following the training, reporting to doctors has also become easier. - The content was satisfactory, and the two trainers were supportive and readily available for discussions whenever any confusion arose. - The training content and WHONET are well-suited for hospital settings, allowing the generation of both microbiological and clinical data. Every three months, we are required to submit an antibiotic report to doctors, and WHONET/BacLink has been very helpful in this process. - The training was effective in terms of both content and methods. PowerPoint presentations and real-time data demonstrations were conducted using hospital data as dummy data for WHONET. However, the training duration was inadequate. Two days were not enough, and there wasn't sufficient time to discuss the real challenges we faced when using the system in a hospital setting. - The training enabled the hospital system to make effective use of WHONET for data recording, reporting, and dissemination. About a month after the training, we began using WHONET and BacLink all the data had been extracted once and cleaned in an Excel file. The IT personnel extracted the data from the system we were using and ensured it was thoroughly cleaned. - The cleaned Excel file was then uploaded into WHONET for analysis, report generation, and submission to clinicians to assist in making decisions about antibiotic prescriptions. - Before the training, Excel was the primary tool for AMR data analysis. The hospital had an inbuilt customized LIS system. Prior to using the customized software, a system called "Worldwide" from the US was used, which is still in operation. However, WHONET is now more commonly used for analysis and report generation. Since adopting WHONET, they have been reporting to NPHL, but not through WHONET or BacLink; instead, they use Excel. The report was submitted last year, but no SQLITE file was sent to NPHL. - The trainers did not mention uploading the file into the GLASS system; this information was later learned through NPHL training. - WHONET is used solely for internal purposes, including data entry, internal analysis, and reporting within the hospital. | 25^th^ July 2022 Interviewee 1Site 1 | |
| **Implementation experiences** |  | |
| - BacLink was implemented in 2020, although WHONET had been in use since 2014. - Every three months, lab personnel analyze antibiotic-related data and present or brief the details to the doctors. - The report submitted to doctors helps them make decisions regarding drug prescriptions. - A codebook for using the codes for the variables to be uploaded has been prepared, although a user manual has not been developed. - She feels that "We haven't been able to fully utilize WHONET software because everyone in positions of authority at the hospital is occupied with their own tasks, and no effort or time has been dedicated to improving or enhancing the current knowledge and use of WHONET. I believe WHONET has far more potential than we're currently using. Additionally, I’ve been so busy that I haven't been able to allocate time to read the system's 'Help' guide." - No antibiotic alerts or breakpoints have been set in the laboratory, and AMR pattern identification is not practiced. Only RIS mapping is performed periodically for each antibiotic against the organism. | 25^th^ July 2022 Interviewee 1Site 1 | |
| **Barriers to implementation** |  | |
| - A technical issue occurred in the system, where multiple alerts and error warning signs appeared on the screen, preventing us from using the system for about a month. - An event organized at X soon provided the opportunity to connect with Dr. A, with whom I consulted about the problem, and he resolved the issue. Since then, there have been no further issues with the system, and we have been using it efficiently. - There is a perceived need for a designated person to consult or discuss the technical problems we encounter while using the software. | 25^th^ July 2022 Interviewee 1Site 1 | |
| About WHONET/Baclink training |  | |
| - The training was conducted prior to COVID. - The training was completed in less than a day. It was more of a brief orientation, a workshop to present the AMR data they had collected from us, rather than a comprehensive training session. - Have been using Midas software. The idea of linking the two systems was discussed, but they couldn't adapt to using WHONET. - A total of six participants attended the training. - Only two of the participants remain at the hospital. - One of the staff members had also participated in a separate WHONET training course held at a hotel for one week, with Dr. B and another technician. This took place before the COVID outbreak, around 2017-2018, at Y hotel. - The trainers who visited the hospital extracted AMR data from Midas and demonstrated how to upload that Excel data into WHONET. However, no practical training was provided at all. - No detailed training was provided on how to extract or enter the data. - The software had been installed in our hospital prior to this training, and it was done through another source, not as part of this training. - It would be highly effective if proper implementation were carried out with the necessary guidance from experts. - Information on data analysis in WHONET was provided, but there was no clarity on the methods or ways to analyze the data within WHONET. - There was no knowledge about how to print reports from WHONET. - The data, once entered, needs to be re-entered into the lab system for Midas. Departments like Emergency, OPD, and ICU have their data linked, but it is not integrated across the entire hospital. - Currently, after billing, patient information or ID can be easily accessed in the lab as well. | Interviewee 2 Site 2  4^th^ August | |
| **Implementation experiences** |  | |
| - WHONET has not been implemented. - I had been eager to get involved in AMR data and had even applied for it at NPHL. - However, no follow-up has been done since then. - After the lockdown, there was a lot of discussion about switching to a different system, so I have been waiting without any action. - There has not yet been a formal study on medication distribution based on the reports, and there is no established channel or mechanism for monitoring. - There are no trained personnel available to guide the use of the system. | Interviewee 2 Site 2  4^th^ August | |
| **Barriers to implementation** |  | |
| - I reached out to the NPHL team for assistance in guiding us on how to use their system. Someone was supposed to be sent to help, but that never happened. - Due to the lack of guidance, they were unable to start working with either the AMR data or WHONET. - During the two years of COVID, there was no activity in the academy, so everything was done online from home. - No follow-up was conducted after that. - There was no expert guidance provided for teaching the staff how to use the software. - There was a change in management. - There was a lack of support from the relevant experts and management. - There was no discussion with the staff regarding the implementation of new software or the introduction of another system. | Interviewee 2 Site 2  4^th^ August | |
| **Way Forward** |  | |
| - Moving forward, if a technical expert comes and provides proper training for the staff over 2-3 days, the system can be effectively used in the hospital. - A discussion with the internal management team can be held, though the need for additional manpower could pose a challenge. - If WHONET were implemented in the hospital, accessing data would be easier, allowing for monthly or yearly calculations, policy development, and more. - Developing a local guideline would also be more straightforward with WHONET. | Interviewee 2 Site 2  4^th^ August | |
| **About WHONET/Baclink training** |  | |
| - There were around 15 participants in the training, with two from our hospital. - We had not used WHONET prior to the training. - If used solely for uploading data, WHONET is definitely easier, but since we use another software for reporting and other tasks, WHONET ends up being used for repetitive work. - The other software we’ve been using for the past year is called Danfe Health. Before that, we were using Midas software. - The training lasted for five days, where both WHONET and BacLink were discussed (likely in Kathmandu), with an additional one-day training at the hospital itself. - WHONET is very useful for data analysis, but it is not fully effective for the hospital as a whole. This is because the hospital software operates separately from WHONET, making it a repetitive and double effort. - During the training, everyone used personal laptops, and it was a bit challenging to keep up with the pace of all participants simultaneously. - It was difficult to get familiar with the terms and terminology used during the training. While medical terms were understandable, IT-related words and other technical aspects were not as easy to grasp. - We were provided with artificial data for practice during the training. - We have completely stopped using WHONET for now and are only using Danfe. - Since patient data can only be uploaded through the backend in WHONET, and Danfe contains all of our patient data, using Danfe was the most convenient option. | Interviewee 3 Site 3  27^th^ July | |
| **Implementation experiences** |  | |
| - It has been used after downloading. - Approximately one year’s worth of data (from 2018-2019) was uploaded to WHONET. - Real-time implementation was not carried out. - Currently, reporting to NPHL is done through Danfe, but it still lacks some of the features we need. Discussions about upgrading or making changes to the system are still ongoing. - NPHL provides us with a format, which we fill out with the necessary data and send to them every month. - No codebook has been created. - Since we started directly uploading data into WHONET, the Baclink has not been used, so we did not focus on any issues related to it. - The confusion primarily revolves around using the Baclink. | Interviewee 3 Site 3  27^th^ July | |
| **Barriers to implementation** |  | |
| - WHONET could not be continued during duty rotations. - There is still a lack of confidence in using the Baclink, and more training or expert assistance is needed. - There is a lack of practical knowledge. - Extracting data and reusing it through the backlink remains difficult. - After COVID, duty kept changing, and the software could not be maintained. - The training on how to use WHONET was not passed on to other staff members. - The financial burden is a concern, as the expenses are covered by the hospital. - The microbiology staff are not very efficient in using such software and systems, and there is a need to involve IT staff in these trainings as well. - The daily workload in the microbiology department is constant, and on top of that, the additional task of uploading data into different systems is not easy. - Since the training was conducted 2-3 years ago, we are bound to face some challenges. | Interviewee 3 Site 3  27^th^ July | |
| **Way Forward** |  | |
| - While it may not be particularly useful for the hospital, it could be valuable for collective data analysis across different countries. - It would be more beneficial if trainers came on-site and provided training for 2-3 days. - It would also be helpful if someone from the IT department participated in the training, so that in case any issues arise, we can seek assistance from the IT staff as well. - The solution we have been discussing is to find a way to use Danfe for uploading and extracting AMR data while linking it to WHONET and other systems, so everything can be handled through the same software. - If training focused specifically on the baclink was provided, it would be possible to effectively use WHONET. | Interviewee 3 Site 3  27^th^ July | |
| About WHONET/Baclink training | |  |
| - The training consisted of two days at the office, with 3-4 hours each day. - Online data analysis training was provided later. - During the training, they covered how to use WHONET, including a demonstration on how to upload and use real data. - Two participants from the hospital attended the training. One staff member is still present, while the other has retired. - I had no knowledge about the selection process for staff attending the training. - We learned about report printing, lab usage, and data analysis from the WHONET system. - The WHONET training was generally good, but we were not able to use it effectively. Currently, we just enter data into our system and print reports, but if we had fully utilized WHONET, we would be able to analyze data from one or two years and generate reports. - The two-day training was sufficient for understanding, but over time, the information fades, and we forget things, making it difficult to resolve confusion when it arises. - WHONET is still installed on the laptop. - With proper training and follow-up, the WHONET system could be implemented in the hospital. - No specific formats were created after the training to help implement WHONET or to train other internal staff. - We were taught how to extract an Excel file and upload it through the backlink in WHONET. - The online training for data analysis in WHONET mainly demonstrated how it could be effective. | | Interviewee 4 Site 4  8^th^ August |
| **Implementation experiences** | |  |
| - Currently, the Dolphin software is being used, which was also in use before the training. - No internal training has been provided to other staff members about WHONET. - The lab uses Dolphin software, and the hospital has another software system, both of which are linked. When the patient ID is entered, all the lab reports are displayed. A digital system and LIS have been implemented. - The WHONET training was overall helpful, but we haven't been able to use it effectively. Right now, we just enter data into our system and print reports. If we had used WHONET, we would have been able to analyze data from one or two years and generate reports. - Analysis cannot be performed in the Dolphin system. If we extract the data and analyze it in another system, it is possible, but calculations cannot be done within Dolphin itself. - I’ve tried extracting an Excel file from Dolphin and analyzed the data with WHONET once or twice before, but now there's been too much of a gap. - I have not communicated the benefits of WHONET to the hospital's management team. - NPHL sends us a quality control sample, and we send them graph reports. Only graph reports are sent; no other samples are provided. - NPHL provides feedback on the reports. - We maintain a list of antibiotics used. | | Interviewee 4 Site 4  8^th^ August |
| **Barriers to implementation** | |  |
| - The microbiology department had only one staff member, and the workload was overwhelming, which prevented the implementation of WHONET. - When confusion arises, we get stuck. Revisiting the training could help make it possible to use the system. - If follow-ups had been conducted by both CAPTURA and hospital management, it would have been more effective, especially if we could have contacted someone for assistance when we faced confusion. - There is a lack of confidence in using the WHONET system due to the gap in experience. - The department is also facing a shortage of human resources. - More effort is needed in approaching the management team to push for the implementation of WHONET. | | Interviewee 4 Site 4  8^th^ August |
| **Way Forward** | |  |
| - If data extracted from Dolphin can be uploaded into WHONET on a monthly basis, it would be effective for data analysis. - Using WHONET to send reports to NPHL would likely bring some changes, but these can be adjusted and integrated accordingly. - Given the current increase in human resources, if training is provided again with consistent follow-ups, WHONET can be successfully implemented. - A tentative plan should be developed outlining what actions need to be taken by the hospital or the WHONET team. | | Interviewee 4 Site 4  8^th^ August |
| About WHONET/Baclink training | |  |
| - The training was conducted for one or two days at the hospital. - There were four participants in the training, but only one is left at the hospital. - The training included practical demonstrations on how to enter data. - Overall, the training was useful and good, and the reporting process was easy. - The trainers did a good job explaining the system, though some aspects were left out during the training. - We were given a contact number for support, and while there haven’t been major issues, I would reach out to someone from NPHL if any problems arise. - No internal training has been provided regarding WHONET. - WHONET was used 3 to 4 days after the training. - Before the WHONET training, data was entered manually into the computer, and I’m not aware of any other software used previously. - Data analysis was taught during the training. | | Interviewee 5 Site 5  5^th^ August |
| **Implementation experiences** | |  |
| - Using WHONET for all clinical data has been highly beneficial. It is used to send reports to NPHL and FHI. - Data analysis is also performed using WHONET. - WHONET is currently in use. - Only one staff member is responsible for all data entry and backlink work in WHONET. - While there are staff members for lab data, the microbiology department only has two staff members. I trained the other staff on how to enter data in WHONET, but I handle everything else. - All data from 2020 to the present has been uploaded. - WHONET is used for sending monthly reports, but another software, Cogent Health, is used in the lab to generate reports. - I used WHONET for six months without using any other software. - Before using WHONET, I manually entered data into a format on the computer to print reports. - The other software lacks the proper format required for sending reports to NPHL. - Isolate listing for data analysis is done, along with quick analysis and RIS percentage. - There have been no significant problems encountered while using WHONET. - No codebooks have been created with instructions on how to use WHONET. - WHONET has not been used to determine sensitive or resistant antibiotics prescribed to patients. | | Interviewee 5 Site 5  5^th^ August |
| **Barriers to implementation** | |  |
| - The report printing from WHONET software was not formatted well, so it is not used for printing reports. - Using two software programs leads to double work and increased workload. - It would be better if the entire hospital system were linked with WHONET. - I have very limited knowledge about baclink, so it is not used. - Codebooks are needed to train new recruits on how to use the software. | | Interviewee 5 Site 5  5^th^ August |
| **Way Forward** | |  |
| - If the format and structure of WHONET were improved, it could become easier to use. - It should be able to link with the admin system to avoid double entry of patient details (e.g., age, name). - If the entire hospital system were connected to WHONET and reports could be generated directly, we would use WHONET fully. - Organizing training sessions and ensuring consistent follow-ups for staff is essential. - If Cognent Health software can be used to send reports to NPHL and is integrated with all systems, WHONET might be discontinued. It cannot be kept only for data analysis because double entry would still be a hassle. | | Interviewee 5 Site 5  5^th^ August |
| About WHONET/Baclink training | |  |
| - There were 3 or 4 participants from the microbiology department in the training, but only 2 of them are still at the hospital. - The training lasted for 4 days and was held in the hospital’s microbiology lab. - During the training, we were taught how to use the software, obtain results, and work with the backlink feature. - I don’t remember much from the training. - While the software could have been useful, it has not been practically implemented, so WHONET hasn’t been very effective. - The training initially seemed useful for microbiology and analyzing bacteria, but it was never put into practice. - Since the training, we have never used the software. - We are currently using Midas software at the hospital. - Midas has recently upgraded its settings, but so far, we haven’t encountered any major issues with this software. - I’ve forgotten about the WHONET system since the training was conducted years ago. | | Interviewee 6 Site 6  8^th^ August |
| **Implementation experiences** | |  |
| - The software has not been used in our hospital. - We have not approached management about it. - All departments in the hospital are using Midas software. - Reports are printed, filed in envelopes, and sent to NPHL following their specified format. - NPHL provides us with the sample and the format for reporting. - A separate list of antibiotics used by the hospital for different bacteria has been created. - Our microbiologist prepares a distinct format for the use of each antibiotic. - If there are issues with the Midas software, we go to the lab in-charge who oversees the system, and then the in-charge contacts the Midas team for assistance. | | Interviewee 6 Site 6  8^th^ August |
| **Barriers to implementation** | |  |
| - Instead of having software dedicated solely to microbiology, the hospital requires a comprehensive integrated system. - If the hospital installs WHONET software, another software will still be needed, leading to double the workload and expenses. - The current human resources are limited, resulting in an overload of work. | | Interviewee 6 Site 6  8^th^ August |
| **Way Forward** | |  |
| - No exact reason or commitment can be given until WHONET is properly used, as it has only been used from the microbiology perspective so far. - If WHONET is implemented in the future, the billing and reporting processes would need to be simplified. - It would be beneficial if future training sessions could include all departments, not just microbiology. - Including the LIS system in the software would improve its functionality. - Having a software expert onsite at the hospital would be ideal, but hospital management may not be receptive to this idea. - The software team may need to consider assigning dedicated staff to each hospital. | | Interviewee 6 Site 6  8^th^ August |
| About WHONET/Baclink training | |  |
| - Various hospitals gathered for AMR surveillance data collection, and during this event, we were introduced to WHONET and baclink as well. - The session was organized by NPHL. - I was the only participant from B Hospital and also the only microbiologist there. - We were given an orientation on data entry, where a demo was shown, but practical training was not provided. - I tried using the system myself a few times by downloading data and manually entering antibiotics. - Although we had been using CNC and didn’t necessarily need WHONET, the training still provided us with useful knowledge - WHONET can be used more effectively from a microbiological perspective. - Data from our hospital has been reported to NPHL annually, and if done through WHONET, it would have been easier. However, frequent software changes made its implementation challenging. - I’m not sure about the name of the software currently being used in the hospital. - A new software was installed just 15 days ago. - I have no knowledge about data analysis using WHONET software. - We were only given a basic orientation on how to use WHONET software. - I have not been trained on the WHONET backlink either. - The main training focused on AMR, and WHONET was only briefly mentioned. | | Interviewee 7 Site 7  8^th^ August |
| **Implementation experiences** | |  |
| - Our hospital frequently changes the software in use, and we only receive basic training each time. - I was using Medipro when I attended the WHONET training. - I haven’t yet pursued the implementation of WHONET software in the hospital. - WHONET required individual data entry, while the software we were using allowed us to link records once entered and print them. - Medipro doesn’t support data analysis. - We will continue using the current software, as it provides patient data and links it across all departments. With the patient ID, we can access their entire profile and records for tests in various departments. - Even though training was provided, not all locations have been using WHONET. - Data from our current software can be converted into Excel and then reported to NPHL. - One issue with Medipro software was that we needed to print separate reports for each type of growth observed. Multiple growths required multiple reports. - There were some billing issues with Medipro. - The newly implemented software also has minor issues, but the team resolves them whenever they arise. The software is not yet fully complete, so we call them whenever a problem occurs, and they come to fix it. - We have an internal IT team, and the software team has a regional office in the valley, so they are available to assist whenever needed. | | Interviewee 7 Site 7  8^th^ August |
| **Barriers to implementation** | |  |
| - Data from our hospital has been reported to NPHL annually. Using WHONET would have made this process easier, but frequent software changes made its implementation challenging. - The other software we used allowed all data to be linked once entered, making it easier for us. - The training provided on WHONET was not enough for proper implementation in the hospital. - We can extract microbiology department data separately from WHONET, but it doesn't link to the overall records. - Only one staff member is trained to use WHONET, so if that person is absent, no one else can enter data. - The decision to change software or implement specific software rests with the management. - Medipro software was removed due to the lack of support from the team in resolving issues. | | Interviewee 7 Site 7  8^th^ August |
| **Way Forward** | |  |
| - If we had received proper training for WHONET, we could use it as a backup during software crashes. - If WHONET is introduced in the hospital, it would be kept as a secondary option. - There is a possibility of conducting internal training if proper WHONET training is provided to a few hospital staff members. - There have been discussions about the government trying to implement the same software across all government hospitals, so if it becomes mandatory, WHONET could be installed permanently. - We have an IT staff who handles all PCR data, so if the person responsible for data entry receives training, it would be beneficial. - If another training is organized, participants from other departments (especially data entry) should also be included, not just lab staff. | | Interviewee 7 Site 7  8^th^ August |
| About WHONET/Baclink training | |  |
| - The two-day training had six participants from our hospital's lab. - During the training, we had to download two things (though I don't recall exactly), WHONET and baclink. - They demonstrated how to upload and enter data into the software using our hospital's data. - The trainers answered our questions and explained things clearly. - Since we haven't really used the software, we don't know much about it. - We were informed about data analysis features in the WHONET software during the training. | | Interviewee 8 Site 8  10^th^ August |
| **Implementation experiences** | |  |
| - Data is entered manually, and although we have an LIS, it hasn't been implemented in microbiology yet. - The hospital is currently using Sukra software, but it is only used for reporting. - The hospital has not been reporting to NPHL up to this point. - The current software is used solely for report dispatch and cannot perform data analysis. - We have been working on data analysis in the hospital, and it is still under discussion. - There shouldn't be much of an issue with data analysis in the future. | | Interviewee 8 Site 8  10^th^ August |
| **Barriers to implementation** | |  |
| - There is a shortage of staff to implement the software, and the microbiology department has limited personnel (with a recent change in the past month to allocate separate staff for microbiology). - Currently, there is no time to focus on other matters, so WHONET as an alternative hasn't been discussed with the in-charge. - The hospital staff is insufficient. - The training was conducted quite a while ago, and I don't recall the details clearly. | | Interviewee 8 Site 8  10^th^ August |
| **Way Forward** | |  |
| - To implement WHONET in the hospital, one full-time staff member will be required.. | | Interviewee 8 Site 8  10^th^ August |
| About WHONET/Baclink training | |  |
| - Recently attended a one-day event at Y Hotel. - Did not personally attend the training. - Was informed about WHONET software by the staff who participated in the training. - Have never used WHONET software but am aware of its benefits for microbiology. - Out of the three staff members who received the training, one has already left the hospital. - After a year or more, the staff may not fully remember how to use the software properly. | | Interviewee 9 Site 9  12^th^ August |
| **Implementation experiences** | |  |
| - The hospital is currently using Midas. - There is no ongoing discussion about changing the software or adding a second one. - The staff who received WHONET training did not use it after the training. - Reports are being sent to NPHL. - The current system links all departments of the hospital, even when work is done separately. - A new lab has been set up. - Midas has been functioning well for all these years, so I haven't given much thought to it. However, I don't know much about WHONET, so I can't compare which one is better. - I don't encounter many issues with Midas. The Midas IT team helps us out whenever there are any problems, though I don't know much about the technical aspects. | | Interviewee 9 Site 9  12^th^ August |
| **Barriers to implementation** | |  |
| - Midas software has been in continuous use since the start. - The initiative needs to come from the administration level. - The current system connects the entire hospital, and it might not be as straightforward with WHONET. | | Interviewee 9 Site 9  12^th^ August |
| **Way Forward** | |  |
| - It would be beneficial if the administration looks into it and works towards implementing the software. - It would be helpful if the lab staff bring up the issue of using WHONET to the management. - Another training session is needed. - I will inform the administration about WHONET for further discussion. | | Interviewee 9 Site 9  12^th^ August |
| About WHONET/Baclink training | |  |
| - There was a previous doctor in charge who had attended several trainings before. - This was the first training attended after being assigned to the microbiology lab. - I was the only person from the microbiology lab at Z Hospital to have received the recent training. - Dr. C had also attended a similar training previously. - I attended a training at Hotel Y on the use of Ms. Excel. - The facilitators explained how to operate Ms. Excel. People from FSI had conducted a structured class at Z Hospital. - I did not attend the WHONET/backlink training, so I have no knowledge of how to use the software. | | Interviewee 10 Site 10  15^th^ August |
| **Implementation experiences** | |  |
| - The hospital is currently using MIDAS software instead of WHONET. - Prior to July 2022, WHONET was being used. - The switch to MIDAS occurred because the data in the microbiology lab has now been digitalized. Before that, the lab did not have its own software, so WHONET was used. - WHONET was only used for data entry. - Monthly reports were submitted to NPHL. - A year's worth of data was entered into the system. - The data consistency across all variables has been maintained. | | Interviewee 10 Site 10  15^th^ August |
| **Barriers to implementation** | |  |
| - WHONET is only used in microbiology labs and not in other departments. - Using both WHONET and MIDAS would involve repeated tasks and double data entry, so the hospital currently only uses MIDAS. - WHONET restricts access to patient details and reports to only the assigned users. - WHONET has not been used for data analysis yet. - The software is not very user-friendly. - There is a lack of motivation among hospital staff and lab officials to conduct their own data analysis. - There is insufficient knowledge about the software. - The printed reports from WHONET are not clear; this issue was raised but was not addressed in any of the training sessions. | | Interviewee 10 Site 10  15^th^ August |
| **Way Forward** | |  |
| - Lab technicians need to be encouraged to perform data analysis on their own, in addition to sending data to NPHL. - If everyone is trained in data analysis using WHONET, its use will become more consistent across hospitals. - With sufficient knowledge of WHONET, it would be possible to persuade hospital management to use WHONET in the microbiology department instead of MIDAS. - A dedicated staff member should be assigned solely for data entry and analysis for both WHONET and MIDAS. | | Interviewee 10 Site 10  15^th^ August |
