# Supplementary material for: Barriers and perceptions of WHONET/BacLink adoption in Nepal: A qualitative study of clinical microbiology laboratories
Source: PLoS One. 2025 Jul 1;20(7):e0326658. doi: 10.1371/journal.pone.0326658 (PMC12212552; doi:10.1371/journal.pone.0326658)
Supplement: Attachment — Submitted filename: Review feedback-PONE-D-24-53694.docx [file pone.0326658.s005.docx]

This manuscript, *"End users’ perception and challenges to adopt the WHONET/BacLink system in clinical microbiology laboratories in Nepal,"* has potential for publication particularly in the context of global health, antimicrobial resistance (AMR) surveillance, and digital health interventions.

Although it presents a real-world evaluation of WHONET/BacLink and provides actionable insights into barriers to software adoption, however this manuscript has some key gaps that need to be addressed. Fo example,

- The title and abstract should be more explicit about the study's qualitative nature. Consider modifying the title to:
  *"Barriers and Perceptions of WHONET/BacLink Adoption in Nepal: A Qualitative Study of Clinical Microbiology Laboratories."*
- The introduction should explicitly state the research question(s).
- The discussion section should have a more structured approach, aligning findings with solutions.

**In methodology,**

- the sample size (10 hospitals out of 19) is small, which limits generalizability. A discussion of this limitation and possible biases (e.g., response bias in phone interviews) is needed.
- The inductive coding approach used for qualitative analysis should be described more transparently (e.g., how themes were validated).

**Data utilization & interoperability solutions**

- Line 15-18: Despite given extensive training on uses of WHONET/BacLink, why do you think lack of knowledge is the barrier?
- The paper extensively discusses barriers but does not explore technical solutions to WHONET/BacLink's interoperability issues. Are there existing case studies or proposed solutions in other countries?
- The manuscript does not sufficiently compare hospitals that adopted the system vs. those that did not—are there contextual factors that influenced successful implementation?
- The study is valuable but lacks a broader policy or global perspective—how do these findings compare to other low-resource settings?
- Some findings (e.g., hospital-specific WHONET usage patterns) would be better presented in tables or figures for clarity.
