# Supplementary material for: Barriers and perceptions of WHONET/BacLink adoption in Nepal: A qualitative study of clinical microbiology laboratories
Source: PLoS One. 2025 Jul 1;20(7):e0326658. doi: 10.1371/journal.pone.0326658 (PMC12212552; doi:10.1371/journal.pone.0326658)
Supplement: Attachment — Submitted filename: Response to Reviewers_editor.docx [file pone.0326658.s006.docx]

PLOS ONE

San Francisco, CA 94103

USA

Dear Editor,

We appreciate your follow up comments on our submission. I am pleased to re-submit our revised manuscript, titled “**“Barriers and perceptions of WHONET/BacLink adoption in Nepal: A qualitative study of clinical microbiology laboratories”** for consideration in *PLOS ONE*.

I have ensured that our manuscript follows the PLOS ONE submission guideline. Furthermore, all the follow up comments from editor have also been addressed in this revised manuscript with following amendments

- **Data Sharing**

**Response**: We have uploaded deidentified and anonymized data as supporting information file in the system.

- It appears that your ORCiD iD has not been validated in your Editorial Manager account, and we are unable to proceed until that step is complete.

**Response**: The ORCiD iD is now validated to the Editorial Manager Account.

Thank you for considering our manuscript for publication.

Sincerely,

**Sanjay Gautam, PhD**

International Vaccine Institute, Seoul
Sanjay.Gautam@ivi.int
02.04.2024
